# Supplementary material for: Clinical impact of heterogeneously distributed tumor-infiltrating lymphocytes on the prognosis of colorectal cancer
Source: PeerJ. 2024 Jan 9;12:e16747. doi: 10.7717/peerj.16747 (PMC10785792; doi:10.7717/peerj.16747)
Supplement: Supplemental Information 1 [file peerj-12-16747-s001.zip › Clinical data-codebook.docx]

1. For CEA

1 denotes negative (serum level <5 **ng/ml**); 2 denotes positive (serum level >5 **ng/ml**)

1. For CA199

1 denotes negative (serum level <37 **U/ml**); 2 denotes positive (serum level >37 **U/ml**)

1. For tumor size

1 denotes tumor size< 5cm; 2 denotes tumor size> 5cm

1. For Lymph node invasion

1 denotes absence of lymph node metastasis; 2 denotes present of lymph node metastasis

1. For Metastasis

1 denotes absence of distant metastasis; 2 denotes present of distant metastasis

1. For chemotherapy

1 denotes patient with chemotherapy; NA denotes not available treatment information
