# Supplementary material for: Clinical impact of heterogeneously distributed tumor-infiltrating lymphocytes on the prognosis of colorectal cancer
Source: PeerJ. 2024 Jan 9;12:e16747. doi: 10.7717/peerj.16747 (PMC10785792; doi:10.7717/peerj.16747)
Supplement: Supplemental Information 2 [file peerj-12-16747-s002.docx]

**Table S1.** Correlations between TILs at the tumor stroma and clinicopathological features

|  | **CD3+ TILs** | | **CD8+ TILs** | |
| --- | --- | --- | --- | --- |
|  | **OR**  **(95% CI)** | ***p*-value** | **OR**  **(95% CI)** | ***p*-value** |
| **Age** |  |  |  |  |
| <50 vs. >50 | 0.6 00  (0.221-1.629) | 0.762 | 0.461  (0.167-1.273) | 0.217 |
| **Sex** |  |  |  |  |
| Male vs. Female | 0.735  (0.302-1.790) | 0.804 | 0.484  (0.196-1.193) | 0.510 |
| **CEA** |  |  |  |  |
| Negative vs. Positive | 0.812  (0.331-1.989) | 0.599 | 0.658  (0.267-1.619) | 0.754 |
| **CA19-9** |  |  |  |  |
| Negative vs. Positive | 0.323  (0.079-1.324) | 0.313 | 0.323  (0.079-1.324) | 0.105 |
| **Tumor location** |  |  |  |  |
| Left vs. Right | 0.794  (0.309-2.039) | 0.962 | 1.260  (0.490-3.237) | 0.964 |
| **Tumor size (cm)** |  |  |  |  |
| <5 vs. >5 | 1.380  (0.555-3.429) | 0.186 | 0.725  (0.292-1.801) | 0.495 |
| **Histological type** |  |  |  |  |
| Adenocarcinoma vs. others | 1.336  (0.464-3.852) | 0.499 | 0.748  (0.260-2.157) | 0.802 |
| **T stage** |  |  |  |  |
| T1+T2 vs. T3+T4 | 0.809  (0.225-2.909) | 0.988 | 1.236  (0.344-4.446) | 0.609 |
| **N stage** |  |  |  |  |
| N0 vs. N1+N2 | 0.814  (0.335-1.981) | 0.996 | 1.509  (0.618-3.687) | 0.174 |
| **TNM Stage** |  |  |  |  |
| I+II vs. III+ IV | 0.902  (0.369-2.201) | 0.826 | 1.684  (0.685-4.144) | 0.130 |
| **Histological grade** |  |  |  |  |
| Well/Moderately vs. Poorly | 1.149  (0.408-3.236) | 0.854 | 0.870  (0.309-2.449) | 0.906 |

**Abbreviations:** OR, odds ratio; CI, confidence interval; CEA, carcinoembryonic antigen; CA19-9, carbohydrate antigen 19-9.
